# Evaluation of sleep quality and duration in pregnancy and risk of gestational diabetes mellitus: A prospective follow-up study

**DOI:** 10.37796/2211-8039.1139

**Published:** 2021-06-01

**Authors:** Saloumeh Peivandi, Ali Habibi, Seyed Hamzeh Hosseini, Mohammad Khademloo, Mohammad Raisian, Hedieh Pournorouz

**Affiliations:** a Department of Gynecology and Obstetrics, Faculty of Medicine, Mazandaran University of Medical Sciences, Sari, Iran; b Student Research Committee, Faculty of Medicine, Mazandaran University of Medical Sciences, Sari, Iran; c Psychiatry and Behavioral Sciences Research Center, Addiction Institute, Mazandaran University of Medical Sciences, Sari, Iran; d Department of Community Medicine, Faculty of Medicine, Mazandaran University of Medical Sciences, Sari, Iran; e Department of Radiology, Faculty of Medicine, Mazandaran University of Medical Sciences, Sari, Iran

**Keywords:** Sleep wake disorders, Pregnancy, Diabetes, Gestational, Snoring

## Abstract

**Background and objectives:**

Sleep disorders during pregnancy may be linked an increase risk of adverse pregnancy outcomes. This study aimed to evaluate the relationship between sleep quality and duration in pregnancy and risk of gestational diabetes mellitus (GDM).

**Materials and methods:**

This prospective follow-up study was performed on 240 pregnant women with a gestational age between 20 to 24 weeks, who were referred to Imam Khomeini Hospital in Sari from 2018 to 2019 for prenatal care. The sleep quality of all eligible women were evaluated with the Pittsburgh Sleep Quality Index (PSQI). During the 26 to 28 weeks of gestation, 1-hour and 2-hour oral glucose tolerance test (OGTT) was done for all women.

**Results:**

The results showed that women with poor sleep quality had a significantly higher mean BMI and 1-hour and 2-hour OGTT results (P < 0.05). Compared to women with good sleep quality, women with poor sleep quality (PSQI >5) had a greater risk of developing GDM (OR = 2.99, 95% CI 1.77 to 5.06). In women with sleep duration of less than 7 and more than 9 hours, the frequency of GDM was significantly higher than women with the 7–9 hour sleep duration (P < 0.05). Also, the frequency of GDM in women with three or more than three times of snoring in a week (63.44%) was significantly higher than women with once a week (30.61%) (P-value <0.001).

**Conclusion:**

It seems that sleep disorders and poor sleep quality can be a risk factor in developing GDM. Therefore, sleep characteristics should be considered in pregnancy care; especially in women with higher risk of GDM.

## 1. Introduction

Sleep disorders, especially short sleep duration (SSD) and poor sleep quality, are prevalent during pregnancy; due to hormonal changes, anxiety, and physical factors that are associated with pregnancy [1–4]. It is believed that the sleep disorders during pregnancy may be linked with increased risk of adverse pregnancy outcomes, such as intrauterine growth restriction (IUGR), preterm delivery, preeclampsia, prolonged labor, and higher incidence of cesarean section [5–7]. From about the 12th week of pregnancy, difficulty in falling asleep, frequent awakenings, reduced nighttime sleepiness, and reduced sleep efficiency begin, and these issues usually continue until two months after delivery [8–10].

SSD has different definitions, often defined as sleeping fewer than seven hours each night [11, 12]. Also, there is clear evidence that supports the relationship between the risk of developing type 2 diabetes and short-term sleep disorders [13–16]. Additionally, many previous epidemiological studies revealed that SSD increases the risk of developing type 2 diabetes, but there are some studies, with conflicting results, about the relationship between sleep disorders and increased risk of gestational diabetes mellitus (GDM) [6, 17, 18]. In a meta-analysis, the results showed that patients with sleep apnea have a higher risk of GDM. However, the link between GDM and poor sleep quality is unclear [19]. Another meta-analysis revealed that long-term sleep duration is a risk factor for GDM [20].

Considering the high prevalence of sleep problems among pregnant women and the possible relationship between GDM and sleep disorders and the lack of strong available supportive evidence in this area in our population and also according to the racial differences and their impact on the prevalence of disorders associated with pregnancy, this is one of the priorities of public health and there is a need to further studies [21]. Therefore, this study aimed to evaluate the relationship between sleep quality and duration in pregnancy and risk of GDM.

## 2. Materials and methods

### 2.1. Study design

This prospective follow-up study was performed on pregnant women who were referred to Imam Khomeini Hospital in Sari from 2018 to 2019 for prenatal care. This study was carried out after approval by the institutional Ethics Committee (code: IR.MAZUMS.IMAMHOSPITAL.REC.1398.2993).

### 2.2. Participants

Singleton pregnant women aged 18–35 years, with a gestational age of fewer than 28 weeks, based on sonography, were included in the study. Mothers with risk factors for GDM including macrosomia, history of GDM in previous pregnancies, persistent glucosuria, obesity with body mass index (BMI) above 30 in the first trimester, history of smoking and alcohol use, intrauterine fetal death (IUFD), miscarriage, diabetes in first-degree relatives, endocrinopathies such as Cushing’s syndrome, pheochromocytoma, and history of glucocorticoid use, beta-adrenergic agonists and thiazides, risk factors for obstructive sleep apnea, such as acromegaly, hypothyroidism, achondroplasia, and craniofacial disorders were excluded.

### 2.3. Data collection

After being eligible, individuals underwent mental health surveys based on the Goldberg General Health Questionnaire (GHQ-12). This self-reporting questionnaire examines a person’s mental state in the last four weeks [22]. GHQ-12, including 12 questions that have six positive and negative items and the scores of negative items that include “not at all”, “as usual”, “a little more than usual”, “much more than usual” are 0, 1, 2 and 3, respectively. For positive items that contain “much less than usual”, “less than usual”, “as usual” and “more than usual”, scores 3, 2, 1 and 0 are considered, respectively. In this case, the minimum score of the GHQ-12 will be equal to 12. The lower a person’s score, the more mental health patient has, and the higher the score than 12, the more likely it is to be considered a mental health disorder [23]. In our study, women with a score of more than 12 were excluded from the study. At the first visit, womens’ demographic and clinical characteristics such as height, weight, BMI and blood pressure (BP) were recorded. The sleep quality of all eligible women was assessed after the 20th week of pregnancy using the Pittsburgh Sleep Quality Index (PSQI) questionnaire. PSQI is a standard tool for evaluating quality of sleep over the past four weeks. The questionnaire involves seven subscales. The sum of the scores of the seven subscales makes up the overall score of a person’s sleep quality. The validity and reliability of this questionnaire have been previously assessed and confirmed (Cronbach’s alpha 0.83) [24]. This questionnaire has also been used in the Iranian pregnant women’s population [25]. Based on this, participants were divided into two groups with and without sleep disorders. Also, the frequency of snoring in women was examined. Then, a 1-hour and 2-hour oral glucose tolerance test (OGTT) was done at 26 to 28 weeks’ gestation. For OGTT, 75 g of oral glucose in the morning (minimum of 8 hours and a maximum of 14 fasting hours), following a 3-day diet without glucose restriction and unrestricted activity was done. Three blood samples were taken first in the fasting state and then one and two hours after eating 75 g of glucose. For each sample, two cc of blood was taken from the patient. The serum was immediately removed from the patient’s blood sample by centrifugation. Diagnosis of GDM was confirmed if the fasting blood sugar (FBS) and the results of blood glucose level after at one and two hours of glucose intake were greater than 92, 180, and 153 (mg/dL), respectively [26–28].

### 2.4. Sample size calculation

Estimation of sample size was based on 7.3% prevalence of GDM during the 24–28 weeks of gestation [21]. At a level of α = .05 with a power of 0.8, we calculated that it was necessary to enroll 200 eligible women for this study, so in order to allow for a withdrawal rate of 20%, we planned to recruit 240 women (120 individuals with and without sleep disorders).

### 2.5. Statistical analysis

Quantitative data followed a normal distribution. Chi-square and independent sample t-test were used to compare the variables between good and poor sleep quality, and chi-square and one-way analysis of variance (ANOVA) was used to compare sleep duration. Post-hoc tests such as Bonferroni, Games-Howell, and least significant difference (LSD) were also used to more accurately examine this significant difference. Pearson’s correlation analysis was used to examine the relationship between blood glucose levels and sleep duration. To evaluate the association between poor sleep quality and sleep duration (<7, 7–9 and >9 hours) on GDM risk, multivariable logistic regression models were used. P-value of less than 0.05 was considered significant. Also Spearman correlation test was also used to determine the relationship between education and sleep duration.

## 3. Results

A total of 310 pregnant women were examined during the study period. Sixty women did not meet the inclusion criteria (poor mental health), and 10 women were excluded during the study due to lack of cooperation. In total, 240 women (120 with and 120 without sleep disorders) completed the study. The mean age (standard deviation) of pregnant women was 3.81 (28.29) years with the range of 19 to 35 years. The mean BMI was 23.56 (5.12). Most of the women (40%) in the study had a diploma. Only 16 women (6.67%) had a history of multiparity. There was no significant difference in terms of educational level and history of multiparity, BP (systolic and diastolic), and FBS among participants (P > 0.05). Women with poor sleep quality had significantly higher mean BMI and OGTT of one and two hours (P < 0.05). The prevalence of GDM was significantly higher in women with poor sleep quality (P < 0.001). The mean daytime and nighttime sleep duration in the group with poor sleep quality was significantly higher (Table 1).

In this study, women were divided into three groups in terms of sleep (25), which included <7 hours of sleep, 7–9 hours of sleep, and >9 hours of sleep. Table 2 shows the mean plasma glucose levels (FBS, OGTT-1h, OGTT-2h), GDM frequency and sleep duration in pregnant women. The results showed that there was a significant difference between the mean FBS, one-hour and two-hour post-prandial glucose level with the daytime and nighttime sleep duration (P-value <0.05). The results of post hoc analysis using Bonferroni, Games-Howell, and LSD tests showed that the FBS and one-hour postprandial glucose level in women with the mean sleep duration of <7 hours was significantly higher than 7–9 hours of sleep duration (P-value <0.05). There was also a significant difference between the frequency of GDM and the sleep duration (P < 0.0001). The results of Bonferroni-corrected test showed that frequency of GDM in women with sleep duration of <7 and >9 hours was significantly higher than women with the 7–9-hour sleep duration (P-value <0.05). Also, using Spearman correlation test, the relationship between education and sleep duration was examined. Based on the findings, no significant relationship was observed between educational level and sleep duration (r = 0.78, p-value = 0.229).

The results showed a significant weak negative correlation between sleep duration and FBS (r = 0.13, P = 0.04), OGTT-1h (r = 0.18, P = 0.005), and OGTT-2h (r = 0.26, P < 0.0001).

Multivariable logistic regression analysis was used to model the relationship between sleep quality and duration with the risk of GDM (Table 3). As shown in Table 3, women with poor sleep quality (PSQI >5) had a higher risk of GDM compared to women with better sleep quality (OR = 1.139, 95% CI 0.69 to 1.85). Among women who had poor sleep quality, 38 (31.67%) and 79 (65.83%) had a sleep duration <7 and >9 hours, respectively. Compared to women with normal sleep duration (7–9 h), these women had an increased risk of GDM (OR = 1.259, 95% CI 0.84 to 1.88).

The results showed a significant relationship between the frequency of snoring and GDM (P < 0.001). The result of the Games-Howel test showed that women with three or more than three times of snoring in a week (63.44%) had significantly higher frequency of GDM than women with once a week (30.61%) (P-value <0.001).

## 4. Discussion

Numerous studies indicated the effect of poor sleep quality and quantity and greater risk of hyperglycemia [29–32]. It has been previously shown that pregnancy is associated with suboptimal sleep quantity and quality. Most of pregnant women experience reduced sleep duration, especially in the third trimester [33–35]. Although, it has previously indicated that the risk of type 2 diabetes is higher in men and non-pregnant women with sleep disorders, there are relatively limited studies to evaluate the association between GDM and sleep problems [36]. Our study showed that the prevalence of GDM was women significantly higher in women with poor sleep quality. Our hospital is the main referral center in northern Iran, and this was one of the reasons for the higher prevalence of GDM in our study (47.5%). Reutrakul et al. reported that there was no significant relationship between sleep disturbances and GDM [6]. Although this study was not consistent with our study, it was generally consistent with our study of the effect of sleep disorders on increasing GDM risk. Wang et al. evaluate the quality and duration of sleep and the risk of GDM in 28–24 weeks pregnant mothers in China showed that poor sleep quality and sleep duration <7 hours and >9 hours a day increases the risk of GDM [21]. Although this study was consistent with our study, failure to use the standard questionnaire to assess the quality and duration of sleep is one of the weaknesses of this study. In our study, women with abnormal sleep duration (<7 and >9 hours) have almost three times higher risk of developing GDM.

A study by Cai et al. showed that a decrease in sleep duration was significantly correlated with impaired FBS, but sleep duration was not correlated with two-hour OGTT. They reported a higher incidence of GDM in women with sleep duration of <6 hours. They also demonstrated the 1.75 times greater GDM risk in women with poor sleep quality [37]. Herring et al. found an inverse correlation between mean nighttime sleep duration and one-hour OGTT values and a positive correlation between shorter nighttime sleep duration and hyperglycemia [38]. Also, Zhong et al. showed that during early pregnancy, poor sleep quality is significantly associated with an increased risk of GDM [39]. Contrary to these studies, Niroomanesh et al. revealed that higher sleep duration can be associated with the increased risk of GDM [40]. Also, the results of a meta-analysis demonstrated that long sleep duration during early and middle pregnancy is closely associated with increased risk of GDM [20]. Also, another recently published meta-analysis indicated that both short and long sleep duration is associated with increased risk of GDM [41]. One possible explanation for this may be the potential changes of sleep duration during pregnancy. In our study, pregnant women were evaluated during their late pregnancy. It has been previously shown that in early pregnancy women usually are more inclined to sleep, while it reduces during the late gestation [42, 43].

In our study, the mean FBS, one hour and two hour OGTT were significantly higher in women with impaired sleep quality. Also, the mean FBS and one hour and two hour OGTT in women who slept less than 7 or more than 9 hours were significantly higher than normal sleep (7–9 hours). We found a weak negative correlation between sleep duration, FBS, 1-hour OGTT, and 2-hour OGTT. Qiu et al. revealed the 5.56 times higher risk of developing GDM among women with <4 hours sleep duration than women normal sleep duration (9 hours). Of course, these results were obtained without considering the BMI of all participants, which was reduced to 3.23 times by adjusting the BMI to less than 25 [19]. Considering that a BMI>30 is a significant risk factor for GDM [44, 45], as a positive point, we only included women with a BMI<30 in this study. However, our study demonstrated that the risk of developing GDM in women with poor sleep quality was 2.99 times greater than women with good quality of sleep. A study by Jahanpak et al. indicated that the frequency of abnormal glucose challenge test in pregnant women with insomnia was significantly higher than in women with normal sleep duration [46].

There was a significant relationship between frequency of snoring and GDM in the present study. So that the frequency of GDM in women who snorted three times a week was significantly higher than once a week. Qiu et al. indicate an association between frequency of snoring and GDM [19].

## 5. Conclusion

It seems that sleep disorders and poor sleep quality can be risk factors in developing GDM. Therefore, sleep characteristics should be considered in pregnancy care; especially in women with a higher risk of GDM.

## Figures and Tables

**Table 1:** Demographic and clinical characteristics of women with good and poor sleep quality.

Variables	Good (N = 120)	Poor (N = 120)	P-value
Age, mean (SD), years	28.27 (3.91)	28.41 (3.95)	0.781*
BMI, mean (SD)	21.62 (4.37)	23.60 (5.71)	0.003*
Education levels, N (%)			
Under diploma	39 (32.5)	26 (21.67)	<0.001*
Diploma	57 (47.5)	39 (32.5)	
More than diploma	24 (20)	55 (45.83)	
Parity>1, N (%)	9 (7.5)	7 (5.83)	0.605**
BP, mean (SD), mmHg			
Systolic	111.42 (7.42)	113.79 (9.72)	0.034*
Diastolic	70.83 (4.36)	72.31 (5.99)	0.030*
Plasma glucose, mean (SD), mg/dl			
FBS	86.5 (10.87)	89.64 (11.45)	0.030*
OGTT-1h	143.45 (30.84)	159.22 (36.65)	<0.001*
OGTT-2h	120.84 (20.83)	134.13 (28.56)	<0.001*
GDM, N (%)	41 (34.17)	73 (60.83)	<0.001**
Sleep duration, mean (SD), hours	8.09 (0.71)	9.15 (3.23)	<0.001*

GDM: Gestational diabetes mellitus.

* Independent sample T-Test.

** Chi-square.

**Table 2:** Comparison of parity, plasma glucose, and gestational diabetes with sleep duration.

Variables	Less than 7 hours mean (SD)	7–9 hours mean (SD)	More than 9 hours mean (SD)	P-value
FBS/mg/dl	90.87 (12.2)	86.33 (10.79)	89.43 (11.15)	0.039*
OGTT-1 h/mg/dl	164.26 (38.81)	143.86 (30.59)	156.75 (36.25)	0.001*
OGTT-2 h/mg/dl	131.58 (34.41)	120.92 (20.58)	135.73 (25.95)	<0.0001*
GDM, N (%)	24 (63.16)	41 (33.33)	49 (62.03)	<0.0001**

N: Number; SD: Standard deviation; GDM: Gestational diabetes mellitus; OGTT: Oral glucose tolerance test.

* One-way ANOVA.

** Chi-square.

**Table 3:** Associations between the risks of GDM; sleep quality and sleep duration.

Variables	Unadjusted OR (95% CI)	Adjusted OR (95% CI)
Sleep Duration	1.84* (1.37–2.48)	1.259 (0.84–1.88)
Poor sleep quality	1.259 (0.99–1.59)	1.139 (0.69–1.85)

OR: Odds Ratio; PSQI: Pittsburg Score Quality Index. Sleep Duration between 7 to 9 hours was considered as a reference.

* p-value< 0.05.
